# Enterococci: Between Emerging Pathogens and Potential Probiotics

**DOI:** 10.1155/2019/5938210

**Published:** 2019-05-23

**Authors:** Olfa Ben Braïek, Slim Smaoui

**Affiliations:** ^1^Laboratory of Transmissible Diseases and Biologically Active Substances (LR99ES27), Faculty of Pharmacy, University of Monastir, Tunisia; ^2^Laboratory of Microorganisms and Biomolecules of the Centre of Biotechnology of Sfax, Tunisia

## Abstract

Enterococci are ubiquitous microorganisms that could be found everywhere; in water, plant, soil, foods, and gastrointestinal tract of humans and animals. They were previously used as starters in food fermentation due to their biotechnological traits (enzymatic and proteolytic activities) or protective cultures in food biopreservation due to their produced antimicrobial bacteriocins called enterocins or as probiotics, live cells with different beneficial characteristics such as stimulation of immunity, anti-inflammatory activity, hypocholesterolemic effect, and prevention/treatment of some diseases. However, in the last years, the use of enterococci in foods or as probiotics caused an important debate because of their opportunistic pathogenicity implicated in several nosocomial infections due to virulence factors and antibiotic resistance, particularly the emergence of vancomycin-resistant enterococci. These virulence traits of some enterococci are associated with genetic transfer mechanisms. Therefore, the development of new enterococcal probiotics needs a strict assessment with regard to safety aspects for selecting the truly harmless enterococcal strains for safe applications. This review tries to give some data of the different points of view about this question.

## 1. Introduction

In recent years, probiotics are being consumed increasingly. Several studies have shown that probiotics, viable microorganisms, are known for their beneficial health effects in human and animal such as immune system strengthening, metabolic disorder reduction, and feed digestibility improvement [1].

In order to screen and select microbial strains with probiotic abilities, the Food and Agriculture Organisation (FAO) and World Health Organisation (WHO) have established some basic criteria, such as the examination of tolerance to the orogastrointestinal transit, production of antimicrobial substances and antibiotic susceptibility, adherence to human intestinal mucosa, and desired immunomodulation activity [1]. Previously, only lactic acid bacteria (LAB) isolated from human gastrointestinal tract were recommended by FAO and WHO for human use [2]. However, many research studies showed that some strains isolated from animals, fermented or nonfermented food products, could be potential candidates to be used as promising probiotics for humans and animals [2]. Among several microorganisms, LAB are popular as probiotic candidates due to their being generally recognised as safe status (GRAS). Bacteria belonging to the genera* Bifidobacterium* and* Lactobacillus* are more commonly used in the fermented food production. Nevertheless, probiotic potential of several other genera of LAB, such as* Aerococcus, Carnobacterium*, and* Enterococcus*, were also explored, due to their technological advantage in the food industry and their health-promoting properties [3].* Enterococcus*, one of the main genera belonging to the LAB group with nearly 50 species, could include strains that are known to be opportunistic microorganisms causing several diseases in humans [4].

In addition, many recent studies have demonstrated an alarming increase in multidrug resistant enterococci, particularly vancomycin-resistant strains and their ability to acquire and transfer antibioresistance genes and virulence factors [5]. Hence, based on these findings, the use of enterococci as probiotics generates serious concern leading to the need of deep research studies to better understand the pathogenicity of these versatile microorganisms and elaborate urgent and accurate measures to distinguish safe strains and select them as efficient probiotics.

The main aims of this review are to summarise the pros and cons of enterococci in view of their future use as probiotics and discuss their dual and controversial features between opportunistic pathogens or promising probiotics.

## 2. General Characteristics of Enterococci

### 2.1. Taxonomy

Enterococci are Gram-positive cocci that occur in pairs or short chains, nonspore forming, catalase and oxidase-negative, and facultative anaerobic [6, 7]. The genus* Enterococcus* belongs to lactic acid bacteria (LAB) and represents the third-largest LAB genus after* Lactobacillus* and* Streptococcus* with 37 species classified based on phylogenetic assessment using 16S rRNA sequencing and DNA-DNA hybridisation [3]. Indeed, new species have been recently discovered such as* E. thailandicus*,* E. ureasiticus*,* E. pallens*,* E. caccae*,* E*.* cammelliae, E. lactis*, etc. [8–12]; however,* E. faecium* and* E. faecalis* remain the most important enterococcal species. Taxonomically, enterococci were classified separately in 1984 [13] after being described as streptococci. Some authors recommend revising the classification of some taxa because of insufficient differences between them to be described as separate species such as* E*.* flavescens* and* E*.* casseliflavus* or to regroup species due to similar characteristics such as the case for* E. avillorum* and* E. porcinus* [14].

### 2.2. Physiological and Biochemical Traits

Enterococci are mesophilic bacteria that could grow from 10°C to 45°C with optimal temperature comprised between 30°C and 35°C [15, 16]. Also, they are able to grow in a huge range of pH from 4.4 and 9.6 and in hyper salty media with 6.5% NaCl. Traits that differentiate enterococci from streptococci are their abilities to survive after 30 min of heating at 60°C, to grow in broth supplemented with 40% of bile salts and to hydrolyse esculin [17, 18].

### 2.3. Habitat

Enterococci are ubiquitous microorganisms that could be present in different environments such soil, water, sewage and plants. Furthermore, they are known to belong to the commensal microbiota of human and animals [19]. Currently,* E. faecalis* predominates the* Entercoccus* species of the gastrointestinal tract followed by* E. faecium*, then* E. durans*, and* E. hirae* [20–22].

### 2.4. Occurrence in Foods

Enterococci occur in different foods; dairy products (cheeses, raw milk) [23–26], fermented vegetables (olives, fermented sorghum) [27–33], meats, fish, and sea foods [34–38].

#### 2.4.1. Enterococci in Dairy Products

The prevalence of enterococci in milk has been traditionally considered as a result of faecal contamination, but many studies have reported that this occurrence is not always related to faecal contamination [7, 23, 24]. In fact,* Enterococcus* spp. has the capacity of adaptation to diverse substrates and growth conditions. Indeed, enterococci could be present in both raw and pasteurised milk of cow, sheep, goat, or camel [7, 39, 40]. Enterococcal strains examples that have been isolated from raw milk are* E. faecalis* and* E. casseliflavus* [41],* E. lactis* [42],* E. italicus*, and* E. faecium* [43].

Enterococci could also occur in cheeses made from raw or pasteurised milk and were commonly* E. faecium*,* E. faecalis*,* E. durans*,* E. casseliflavus,* and* E. lactis* [41, 44–46]. This prevalence is different among cheeses resulting in cheese type, milk used in the manufacture, production season, and conditions of production, and ripening [47, 48]. Moreover, it is important to denote that* Enterococcus spp*. play a beneficial role in cheese fermentation as well as in cheese ripening and development of specific flavour, texture, and taste probably through proteolytic, esterolytic and lipolytic activities, citrate breakdown and production of diacetyl, and other important volatile compounds [47–51].

#### 2.4.2. Enterococci in Fermented Vegetables

Enterococci can be present in fermented vegetables due to the fermentation reaction with the predominance of* E. faecium* and* E. faecalis* in fermented soya, sorghum, and olives [18, 52–55].

#### 2.4.3. Enterococci in Meat

Since enterococci are part of the commensal microflora of animal gastrointestinal tract, they could thus occur in meat when slaughtering. The common species are* E. faecium*,* E. faecalis*,* E. mundtii*,* E. durans*,* E. casseliflavus*,* E. gilvus, *and* E. hirae* [56–58]. Fermented salamis and sausages could also host enterococci [59, 60].

#### 2.4.4. Enterococci in Fish and Sea Food

Several enterococcal species have been isolated from fish (viscera and skin):* E. mundtii*,* E. faecium,* and* E. durans* [61–66]. Regarding sea food, the prevalence of enterococci is lower than that in fermented or raw fish [67]. The common isolated strains were* E. faecium*,* E. faecalis, E. casseliflavus*, and* E. hirae* [68]. In regard to fresh shrimps, strains of* E. faecium*,* E. faecalis*,* E. lactis*,* E. casseliflavus*, and* E. gallinarum* have been isolated and reported in many studies [69–72].

## 3. Enterocins

### 3.1. Classification

Enterocins are the bacteriocins produced by* Enterococcus* spp. They are ribosomally synthesised, cationic, hydrophobic, and heat stable peptides with small molecular weight containing about 20-60 amino acids [19, 37, 66, 74–77]. They are insensitive to rennet and stable over a wide range of pH values [78, 79]. They are classified into four classes: lantibiotic enterocins (class I) such as cytolysin, nonlantibiotic enterocins (class II) with three subclasses (1, 2, and 3) such as enterocin A (class II-1), enterocin Q (class II-2), and enterocin B (class II-3), followed by cyclic enterocins (class III) such as enterocin AS-48 and enterocins with high molecular weights (class IV) such as enterolysin A [73].  Table 1 represents with details the enterocins' classification. Most of the characterised enterocins belong to the class II.

The hemolytic bacteriocin (cytolysin) and the circular AS-48 were known as* E. faecalis *bacteriocins and were genetically and biochemically well characterised [80–84].

The subclass II.1 represents the largest enterocin subclass which includes the most abundant enterocins of enterococci. These enterocins share the consensus sequence YGNGV in their N-terminal part which is a prerequisite for their antimicrobial activity and particularly antilisterial activity. It is important to note in this context that enterocin A is among the most potent antimicrobial bacteriocin in this subclass [85–89]. Interestingly, enterocin A is known to be coproduced with other bacteriocins, often in combination with enterocin B [90] and occasionally with enterocin P, enterocins L50, or enterocin Q [91, 92]. Hence, enterococci seem to have the genetic capacity to produce more than one enterocin, as commonly observed among some other multiple-producing bacteriocin lactic acid bacteria (LAB) [93–95].

### 3.2. Spectrum of Action

Enterocins produced by enterococci are small antimicrobial peptides known to display broad-spectrum of inhibitory activity against spoilage bacteria and foodborne pathogens [96–99]. Remarkable antimicrobial inhibitions were observed towards* Listeria monocytogenes*,* Bacillus cereus*,* Staphylococcus* spp., and* Clostridium* spp. [71, 78, 79, 83, 97, 100]. Antagonistic activities against Gram-negative bacteria such as* Pseudomonas aeruginosa*,* Escherichia coli*, and* Vibrio cholera*, against fungi and yeasts, as well as against virus, were also observed with enterocins [66, 101, 102].

### 3.3. Mode of Action

Enterocins, as most bacteriocins, have the cytoplasmic membrane as their primary target [103–106]. They form pores in the cell membrane, thus depleting the transmembrane potential and/ or the pH gradient which result in the leakage of indispensable intracellular molecules [107–109]. The mode of action enterolysin A is quite different from the other enterocins because it attacks susceptible bacteria by degrading the cell wall structure, which eventually leads to lysis of the cells of target strains [110].

## 4. Pathogenicity of Enterococci

Enterococci are among the most common nosocomial pathogens that could cause important infections and diseases such as endocarditis, bacteremia, urinary, intra-abdominal and pelvic infections, central nervous system infections, etc. [4]. Among these infections, approximately 80% were associated with* E. faecalis* [111]. Enterococci, previously viewed as microorganisms of minimal clinical impact, have emerged now as common opportunistic pathogens of humans [112].

Traits implicated in their pathogenicity are virulence factors and the increase of antibiotic resistant strains, especially vancomycin-resistant enterococci (VRE) [5, 113, 114]. As a result,* Enterococcus* spp. represent a main challenge to health staff when identified as the principal cause of infection or illness, particularly in immunocompromised patients [115]. Infections caused by enterococcal strains are originated from the intestinal microbiota of the patient and can be transferred from one person to another or can be acquired by the consumption of contaminated food and water [116].* Enterococcus* spp. is capable of transferring the antibiotic resistant genes (ARG) to produce* β-*haemolysis, gelatinase and aggregation substance that are common enterococcal virulent traits [117].

### 4.1. Virulence Factors

A virulence factor is an effector molecule that enhances the capacity of a microorganism to cause illness. Virulence factors of enterococci play a significant role in the pathogenicity of enterococcal strains. These factors have been intensively investigated in the last few years. The most common and well described virulence determinants in enterococci are aggregation substances (*agg*,* asa1*), cytolysin (*cyl*), gelatinase (*gelE*), extracellular surface protein (*esp*), adhesion to collagen (*ace*,* acm*), and adhesion-like endocarditis antigens (*efaAfs* and* efaAfm*) [118].

Aggregation substances (*agg* and* asa1*) are virulence factors inducing surface protein of* Enterococcus* spp. strains which promote aggregate formation during bacterial conjugation and mediate the specific binding to epithelial cells for colonisation and exchange of plasmids carrying virulence traits and antibiotic resistance genes as well [119, 120]. In addition, the aggregation substances could bind to extracellular matrix proteins such as collagen type I, fibronectin, and thrombospondin [3]. Regarding* agg* gene increases the hydrophobicity of the enterococcal surface inducing localisation of cholesterol to phagosomes and delaying fusion with lysosomal vesicules [121]. Up to date,* agg* determinant is exclusively found in* E. faecalis* strains [122, 123].

Cytolysin (or *β*-haemolysin) is known as protein bacteriocin/heamolysin bifunctionality and is the most studied virulence factor in enterococci. It constitutes a peptidic toxin able to lyse cells by forming pores in the cytoplasmic membrane of bacterial target cells [124]. The frequency of death caused by infection due to a cytolysin-producing* Enterococcus* is five times higher than that observed in a noncytolysin-producing enterococcal infection [125]. Studies on endocarditis have shown that there is a synergism between* cyl* and* agg* genes.

Gelatinase is an extracellular Zn-metallo-endopeptidase (EC 3.4.24.30) implicated in the hydrolysis of gelatin, collagen, *β*-insulin, haemoglobin, casein, and other bioactive peptides [126]. Gelatinase is able to cleave fibrin and damage host tissue allowing thus bacterial migration and spread which raise its implication in virulence of enterococci particularly* E. faecalis*[3]. Furthermore, this protease plays an important role in the formation of biofilm which allows enterococci to colonise tissues and persist in some infection sites [126]. It is necessary to mention that some researchers reported that even when the* gelE* determinant gene is detected, a negative phenotype could be found [127, 128].

Extracellular surface protein (*esp*) is a virulent gene determinant associated with the cell-cell adhesion, particularly adhesion to eukaryotic cells and evasion of the immune response of the host [129, 130]. This gene, which promotes colonisation, is located in a highly conserved chromosome region within the genus and is mostly common in* E. faecium* [129, 130].

The adhesion genes to collagen,* ace,* and* acm*, of* E. faecalis* and* E. faecium*, respectively, bind to collagen types I and IV enhancing virulence strains, while* acm* could also bind to laminin [3]. Also, the adhesion* acm* is known to be part of the subfamily of bacterial adhesions surface called Microbial Surface Components Recognising Adhesive Matrix Molecules (MSCRAMM) that adhere specifically to the protein layer of the extracellular matrix of the host [129, 130].

The* efaA* virulence gene is strongly involved in endocarditis [3]. The most known are* efaAfs* and* efaAfm* for* E. faecalis* and* E. faecium*, respectively [131].

Other virulence determinants are less identified in enterococci and not well described that are also implicated in enterococcal infections. Among these virulence factors is* sag* gene secreted by* E. faecium* which was able of broad-spectrum binding to extracellular matrix proteins [132]. Another* E. faecium* adhesion called* scm* could efficiently bind to collagen type IV [133]. Furthermore, the* ebp* gene encoding endocarditis and biofilm-associated pili were observed to enhance biofilm formation in* E. faecalis* [134]. Also, the* bee* gene (biofilm enhancer in* Enterococcus*) was shown to confer a high biofilm-forming phenotype to* E. faecalis* [135]. Finally, a further virulence factor nominated* hyl*, encoding a hyaluronidase, was shown to hydrolyse hyaluronic acid with a possible role in translocation [136]. This virulence factor was shown to be associated with antibiotic resistance genes and pilin genes on the plasmid [137].

In general, the incidence of all of these virulence factors was lower in* E. faecium* strains than in* E. faecalis* strains, and the virulence of enterococci could not be explained only by the presence of virulence determinants; antibiotic resistance genes play an imminent role in the pathogenicity of enterococcal strains [3, 138].

### 4.2. Antibiotic Resistance

Resistance of some enterococci to commonly used antibiotics is another important virulence trait which strongly enhances the pathogenicity of* Enterococcus* spp. by making them effective opportunistic microorganisms in nosocomial infections [139–141]. In fact, continuous exposure to antibiotics and their intensive use in human and veterinary medicines as prophylactic agents or growth promoters, respectively, have provoked increase in the incidence of enterococcal strains resistant to multiple different classes of antibiotics and may be through genetic mutations conferring this antibioresistance of enterococci and enabling their survival. Hence, this drug resistance becomes an important public health concern. Antibiotic resistance in enterococci could be generally produced by target modification, alterations that affect access of the drug to the target or enzymatic drug inactivation [142].

Intrinsic antibiotic resistance of enterococci includes resistance to cephalosporins, sulphonamides, lincosamides, *β*-lactams, and aminoglycosides, located in the chromosomes [130, 143]. Acquired resistances in enterococci from other microorganisms, via plasmids or transposons, could be observed toward chloramphenicol, erythromycin, fluoroquinolones, tetracycline, penicillin, ampicillin, aminoglycosides (gentamicin, kanamycin, and streptomycin) and glycopeptides especially vancomycin [142, 144]. In fact, vancomycin resistance is of special concern because VRE were known to cause serious infections and diseases that could not be treated with conventional antibiotic therapy [63, 145]. So, VRE posed a real challenge to clinicians since this antibiotic has traditionally considered the “drug of last resort” in the treatment of enterococcal infections as it is often used to replace penicillin, ampicillin, and aminoglycosides in patients with allergies [146]. For this reason, new drugs were evaluated as alternative candidates to vancomycin such as quinupristin-dalfopristin, oxazolidinones, everninomycins, and daptomycin [143].

At present, there are six known genes of glycopeptide resistance in enterococci:* vanA*,* vanB*,* vanC*,* vanD*,* vanE*, and* vanG*. The* vanA* type is the most important operon characterised by strains with high levels of resistance to vancomycin and teicoplanin and its main reservoir is* E. faecium* [130]. The* vanB* operon induces several levels of vancomycin resistance but not teicoplanin resistance. Only* vanA* and* vanB* genes have the ability to transfer vertically and horizontally and to confer high levels of resistance [130]. The* vanC* determinant induces low level of vancomycin resistance and intrinsic sensitivity to teicoplanin. The* vanD*,* vanE*, and* vanG* operons encode low to moderate resistance to vancomycin [130]. In general, it is interesting to know that* vanA*,* vanB*,* vanD*,* vanE,* and* vanG* genes are considered to be acquired properties, while* vanC* gene is an intrinsic trait of motile enterococci [130].

On the other hand, several studies performed in European and American countries reported that VRE colonisation occurs in the community besides human reservoir; animal, environmental, and food reservoirs could act as community sources for VRE outside the health care setting [143]. In this context, VRE were detected with* vanA* gene cluster in animal husbandry due to the use of avoparcin as a feed additive [143]. Effectively, in 1975 avoparcin was used as growth promoter in Europe, Australia, and several other countries, but was not allowed in the USA and Canada [145]. Interestingly, high level occurrence of VRE was observed in European animal farms; however, no VRE were detected in animal farms in the US [147]. Thus, the use of the glycopeptide avoparcin for animal growth promotion was prohibited in Europe and as a likely result, there was a rapid decline of VRE in European farms but no a total disappear [145]. Many hypotheses were suggested to explain this VRE persistence; the first one reports the fact that the use of macrolide tylosin could coselect for VR since both the resistance determinants are located on the same plasmid or that plasmid addiction systems could be implicated in the retention of the resistance [145].

Furthermore, VRE could also occur in human outside hospitals confirming that a transfer of resistance genes between animal and human or a clonal spread of resistant strains could explain this prevalence. In addition, VRE could reach foods via environmental contamination from different sources; waste water from sewage treatment, livestock faeces, and manure from poultry farms [143, 148].

Other antibiotic resistant enterococci have been found among food animals and environment worldwide. In fact, high gentamicin-, kanamycin-, streptomycin-, tetracycline- and glycopeptides-resistances have been observed among enterococci (*E. faecalis*,* E. faecium*,* E. casseliflavus*, and* E. gallinarum*) isolated from bovine mastitis (80%), chickens (62-64%), pigs (57%), food of animal origin (e.g., white and red meats), uncooked food (e.g., lettuce), sewage, and water [145, 149–151].

In general, the emergence of this high antibiotic resistance in all of these various reservoirs and environments suggests interstrain transmission of resistance genes.

### 4.3. Transfer of Virulence Factors and AR Genes

Enterococci are known for their genome plasticity [142]. Indeed, they are able to integrate and use some mobile genetic elements like plasmids, transposons, prophages, and insertions sequences allowing them to easily transfer acquired determinants among strains of the same species, or species of the same genus or other pathogenic and nonpathogenic bacteria as well. In this context, enterococcal virulence factors and AR genes are renowned to be associated with some highly transmissible plasmids [127]. Virulence traits and antibioresistance in enterococci were previously reported to be caused by gene horizontal or vertical transfer mechanisms and by ability to receive genetic material [143]. In this context, Coburn et al. [152] demonstrated the horizontal transfer of a 150 kb cluster called “pathogenicity island” (PAI), previously described in* E. faecalis* by Shankar et al. [153] that contain about 100 operons some of which code for virulence genes (toxins, cytolysin, surface proteins, and aggregation). This horizontal transfer of the pathogenicity island was carried by a plasmid in response to pheromones. Regarding resistance to macrolide antibiotics, lincosamides, and streptogramins (MLS), De Leener et al. [154] have demonstrated, through a genetic marker (*ermB*), the horizontal transfer of these AR genes from an* E. faecium* strain of animal origin to a strain of human origin. This mechanism of propagation via the transfer of genetic elements (plasmids and/or transposons) is more important than clonal dispersal of antibiotic resistant strains [155]. These experiments were conducted on animal models and did not take into account the natural environment that strongly influences the transfer of moving elements.

Of concern, transconjugation in which enterococci acquired virulence and AR determinants could represents a real risk to a safe enterococcal strain that is free of these virulent determinants could unfortunately acquire such genes in both of human or nonhuman reservoirs which raises serious worry regarding their safety for use as probiotics.

## 5. Enterococci as Probiotics

Probiotics are defined as “live microorganisms which when consumed in sufficient amounts, affect beneficially the health of the host.” Health benefits that confer probiotic microorganisms include modulating immunity, enhancing intestinal barrier function, or altering pain perception [1].

Most probiotics are of intestinal origins and belong to the lactic acid bacteria (LAB) particularly to genera of* Bifidobacterium* and* Lactobacillus*, while enterococcal strains are occasionally used [3]. In this context, many studies have been conducted to evaluate the probiotic characteristics of* Enterococcus* strains and clear beneficial and significant health-promoting effects of enterococci were reported [3, 156–160]. Indeed, enterococci were used as probiotics for several purposes and these different applications include pharmaceutical industry, human and veterinary medicines and food industry since some probiotic enterococci could be used in the production of functional foods [1].

In fact, some enterococcal strains such as* E. faecium* M74 and* E. faecium* SF-68 are included as food supplements in several probiotic preparations that have been proved to be effective and safe, such as FortiFlora® and Cernivet® (containing* E. faecium* SF68®, Cerbios-Pharma SA, Switzerland), and Symbioflor® 1 with* E. faecalis* (Symbiopharm, Herborn, Germany) [142, 161, 162].

Enterococcal probiotics can be used in treatment and/or prevention of certain human and animal diseases such as alleviation of irritable bowel syndrome symptoms and antibiotic-induced diarrhea and prevention of different functional and chronic intestinal diseases [163]. Moreover, some enterococci exhibit antimutagenic, anticarcinogenic, hypocholesterolemic, and immune regulation effects [17].

*E. durans* M4-5 has been found to generate butyrate, short chain fatty acids (SCFAs), that induce significant anti-inflammatory effects and contribute to the integrity of the intestinal epithelium [164, 165].

*E. mundtii* ST4SA was recently presented as another potential probiotic strain [166] and* E. durans* KLDS 6.0930 has been postulated as a probiotic candidate through lowering human serum cholesterol levels [167].

More recently, the strain* E. durans* LAB18s was recommended useful for use as a source of dietary selenium supplementation [168], while* E. faecium* LCW 44 and* E. durans* 6HL were shown highly potent against Gram-positive [169] and Gram-negative bacteria [169, 170], respectively.

In feed regulation, the European Food Standards Agency (EFSA) authorised certain strains of enterococci for use as silage additive and dietary supplements. For instance, some enterococcal probiotics were included in the group of feed additives for stabilising the microbial communities of the digestive tract in both monogastric and ruminant animals [171]. Strains of* E. faecium* NCIMB 11181 and* E. faecium* DSM 7134 were approved as feed additives for calves and piglets by EFSA. The probiotics* E. faecium* SF68® and* E. faecalis* Symbioflor 1 are also used to prevent or treat diarrhea in pigs, poultry, livestock, and pets [3]. Furthermore, among the claimed advantages of probiotic enterococci is its positive effects on the performance characteristics of the growth and health of farm animals. In this context, feeding pigs with a probiotic* Enterococcus* spp. was found to reduce intestinal pathogens [172]. Likewise, oral administration of* E. faecium* NHRD IHARA by postweaning piglets has increased serum and fecal IgA levels and improved piglets growth [173]. In chickens,* E. faecium* was demonstrated to improve growth, intestinal morphology, and the caecal microbiota homeostasis [174].* E. faecium* was also reported to enhance the metabolic efficiency and decrease inflammatory responses in broilers [175].

On the other hand, numerous studies have shown the beneficial effects of enterococci in aquaculture. In fact, several works reported a wide spectrum of inhibition by* E. faecium* toward aquatic pathogens including* Yersinia ruckeri, Vibrio harveyi, Streptococcus agalactiae,* and* Aeromonas veronii* [176]. In addition, many trials have investigated the efficacy of* E. faecium* incorporated in feed to improve fish growth and stimulate immune response [177].

Due to safety concerns, lack of safety information, and legislation, only a limited number of enterococcal probiotics are commercialised.* Enterococcus* has not yet obtained the status GRAS [3]. However, some well characterised enterococcal strains are used as starter cultures, cocultures, or protective cultures in food industry and/or probiotics due to their positive attributes. The dual trait of being good candidates as probiotics and opportunistic pathogens of enterococci remains a controversial issue which turns about the question whether enterococci are safe for probiotic use that also remains difficult to answer. The main concern for* Enterococcus* spp. as probiotics is their pathogenicity based on horizontal transfer of virulence factors and AR genes, as explained above, and the increasing number of enterococcal infections in recent decades [1, 178]. Nevertheless, the most important and interesting evidence is that enterococci are not suggested as foodborne pathogens [179]. Indeed, after being suspected of causative agents of foodborne illness in 1926, many studies on enterococci, particularly* E. faecalis* and* E. faecium*, including experiments on animals and volunteer humans were carried out to prove that enterococci cause foodborne illness, but investigations yielded negative results because these bacteria are generally identified in mixed presence with other pathogens such as staphylococci or others [180]. Subsequently, enterococci have emerged as nosocomial- and community-acquired pathogens rather than foodborne pathogens [181, 182]. Still, the safety of enterococci before their use in foods or in probiotic preparations should be carefully assessed. Effectively, when selecting an enterococcal probiotic strain, a number of properties should be considered involving safety aspect and functional and beneficial traits. Since probiotic effect is strain dependent, it should be thus well characterised (phenotypically and genotypically) and must be safe and free of any pathogenicity such as the absence of virulence factors and acquired AR genes [183, 184]. Desirable characteristics for probiotic strain include also the ability to survive and retain viability at harsh gastrointestinal tract conditions of a healthy human (low pH, pepsin, pancreatin, bile salts), their unability to translocate the intestinal mucosa, their susceptibility to phagocytic killing, and the ability to produce antimicrobial substances such as enterocins [1, 183, 184]. Further considerable trait for potential enterococcal probiotics is that they should have limited ability to exchange DNA* in vivo* [1].

## 6. Conclusion

Enterococci are ubiquitous microorganisms that could be naturally present in several food products. Many studies have reported the beneficial effects of enterocin-producing* Enterococcus* strains as starters, adjunct starters, protective cultures, or probiotics. However, very few enterococci have been used as probiotics or feed additives because of the safety concern associated with their pathogenic trait as opportunistic microorganisms capable of causing severe infections and diseases due to their potential virulence factors and antibiotic resistance genes. To date, there have been no reports of disease caused by probiotic enterococci that are currently on the market such the case of* E. faecium* SF68 and* E. faecalis* Symbioflor, which is a great indication of the safety of these enterococcal probiotic strains.

Hence, enterococcal strains in view of future use as probiotics must be well characterised and perfectly assessed regarding safety aspects. For this, modern scientific techniques, up-to-date knowledge of enterococci and their properties, implementation of adequate guidance, and appropriate legislation are strongly recommended to differentiate between pathogenic and safe enterococcal strains and thus could help industrials, health staff, and consumers to accept these strains as potential candidates for useful and beneficial applications as probiotics, like other LAB strains. These measures should be complemented by a more prudent use of antibiotics in human and veterinary medicines and a strict control regarding the presence of enterococci in environmental and food sources to prevent or limit the spread of pathogenic enterococcal strains. Finally, a specific assessment of community transmission is also needed.

Therefore, until now, the debate remains open. In fact, as a coin with two sides, for enterococci, despite their health-promoting properties, they may possess detrimental traits which make it difficult to establish a clear decision within enterococcal strains between emerging pathogens and potential probiotics.

## Figures and Tables

**Table 1:** Classification of enterocins [73].

Class	Sub-class	Sub-group/ Characteristic	Examples
*Class I*	Lantibiotic enterocins	Heamolytic bacteriocins Formed by two peptides cylLs and cylLL Their action needs the presence of the two peptides	Cytolisin

*Class II*, small nonlantibiotic peptides	*II.1* possesses a cationic and hydrophile region with consensus sequence YGNGV in the N-terminal extremity and a disulphide bridge formed by two cysteins in the N-terminal extremity	*Sub-group 1 *possessesan ABC transporter for the secretion of enterocins	Enterocin A, Enterocin CRL35
		*Sub-group 2* The production is realised via a mature pre-protein	Enterocin P, Enterocin SEK4, Bacteriocin 31, Bacteriocin T8
	*II.2* synthesised without leader peptide, did not possess the consensus sequence, nor the system of secretion ABC transporter	*Sub-group 1 *Monomeric proteins	Enterocin RJ-11, Enterocin Q, Enterocin EJ97
		*Sub-group 2* Need for the formation of an heterodimeric complex	Enterocin L50, Enterocin MR10
	*II.3*	Linear enterocins with leader peptide	Enterocin B, Bacteriocin 32 Enterocins1071 A and B

*Class III*, cyclic enterocins		Cyclic peptides	Enterocin AS-48 Enterocin AS-48 RJ

*Class IV*, proteins of high molecular weight		Peptides of high molecular weight (34.5 kDa) and heat-labiles	Enterolysin A
